# Thioacetamide promotes osteoclast transformation of bone marrow macrophages by influencing PI3K/AKT pathways

**DOI:** 10.1186/s13018-022-02938-4

**Published:** 2022-01-29

**Authors:** XiaoLi Jin, Yang Li, Yayang Yang, Hao Shen, Jin Chen, Bin Xu, Jian Xu

**Affiliations:** 1 School of Medical Technology and Information Engineering, Zhejiang Chinese Medical University, Hangzhou, 310053 China; 2 School of Basic Medical Sciences, FUDAN University, Shanghai, 200433 People’s Republic of China; 3 Department of Laboratory Medicine, The First People’s Hospital of Shangqiu, Shangqiu, 476000 China; 4 Department of General Surgery, School of Medicine, Sir Run Run Shaw Hospital, Zhejiang University, Hangzhou, 310016 Zhejiang People’s Republic of China

**Keywords:** Thioacetamide, Osteoclast, Macrophages, RANKL, Cell differentiation

## Abstract

**Background:**

Osteoclast cell increase is a major risk factor for osteoporosis and degenerative bone and joint diseases. At present, RANKL and M-CSF are commonly used to induce osteoclastogenesis. Thioacetamide (TAA) can lead to many types of liver and kidney damage, but less attention has been paid to the association of TAA with bone damage. In this work, we investigated the effects of TAA on the osteoclastogenesis and differentiation of bone marrow macrophages (BMMs).

**Methods:**

BMMs of SD rat suckling mice were taken for primary culture. CCK-8 was used to detect the toxic effects of TAA on BMMs, and flow cytometry was used to detect the effects of TAA on the cell cycle, cell viability, apoptosis and intracytoplasmic Ca^2+^ concentration of BMMs. TRAP staining was used to detect the effect of RANKL and M-CSF and TAA on osteoclast differentiation of BMMs. Western Blot was used to detect the expression level of PI3K/AKT pathway and osteoclast-specific proteins (TRAP and cathepsin K).

**Results:**

The results suggested that TAA inhibited the proliferation of BMMs, while enhancing osteoclastogenesis at 0.5 mg/mL and 1 mg/mL as assayed by TRAP staining. Exposed to TAA, BMMs could differentiate into osteoclast-like cells with overexpression of cathepsin K and TRAP proteins. Western blot results showed that TAA can activate the expression levels of P-PI3K, P-AKT, P-P38, and P-JNK, accompanied by apoptosis of BMMs and increase in intracellular Ca^2+^.

**Conclusion:**

TAA may induce osteoclast formation in BMMs by activating the expression of PI3K/AKT pathway proteins, which is comparable to the classic osteoclast differentiation inducer RANKL and M-CSF. This suggests that we may find a cheap osteoclast inducer.

## Introduction

TAA as a commercial chemical is widely used in electroplating additives, photographic drugs, pesticides and dyeing aids [1–4], and it is used to establish an animal model for liver cirrhosis due to its hepatotoxic effects [5]. TAA-induced cirrhosis has similar pathologic changes to human liver cirrhosis and can be used to reproduce the human disease [6, 7]. Additionally, the effects of TAA are not limited to the liver, as profound structural and functional changes have been described in the kidney, spleen, lung, intestine, stomach, brain and bone marrow after TAA exposure [8–15]. However, less attention has been paid to the association of TAA with bone damage.

In a previous experiment, operation staff found an interesting phenomenon in the classic liver cirrhosis animal model administered with TAA. Approximately, 40 to 50% of the animals presented open fractures, arthroncus and arthrorrhagia. No reports have expressly described bone damage caused by TAA. Lassila and Virtanen [16, 17] found that osteoblastic activity and osteoid were distinctly decreased, and found robust osteoclastic resorption, when the alveolar bone was under occlusal stress and TAA traumatization. Nakano and colleagues [18] also demonstrated that the bone volume in TAA-exposed cirrhotic rats was significantly lower, with a combination of low bone formation rates and high resorption rates.

Bone homeostasis in vivo depends on the balance in function of osteoblasts and osteoclasts, which are responsible for bone formation and bone resorption, respectively [19–22]. These cell populations are tightly coordinated with each other under normal conditions. When the immune function is abnormal, this balance will be broken [23], leading to diseases that affect bone loss, such as rheumatoid arthritis [24, 25], osteoporosis [26] and periodontitis [27].

BMMs possess self-renewal capacity and play a crucial role in modulating normal bone homeostasis, with the potential to differentiate into osteoclasts in vitro [28]. In vitro, receptor activator of nuclear factor kappa B ligand (RANKL) and macrophage colony-stimulating factor (M-CSF) are used to induce osteoclast differentiation [29–31]. Under the co-stimulation of RANKL and M-CSF, the PI3K/AKT pathway was activated, upregulating the nuclear transcription factors NFATc 1 and c-Fos. Then, the expression of the osteoclast-specific proteins TRAP and cathepsin K increase, promoting the differentiation of osteoclast precursor cells into mature osteoclasts [32, 33]. However, RANKL and M-CSF are expensive and the preparation process is complicated, which complicates the study of osteoclasts in vitro. On the contrary, the preparation method of TAA is simple and cheap. TAA may induce osteoclast formation or increase the activity of these cells to cause bone damage. We sought to know whether TAA could induce osteoclastic transformation of BMMs in vitro, so as to find a better osteoclast inducer. Therefore, we conducted experiments to verify the effects of TAA on inducing osteoclastogenesis.

## Methods

### Cells and cell culture

Sprague–Dawley (SD) rats were purchased from Shanghai SLAC Laboratory Animal Co, Ltd. The BMMs were isolated from 2-day-old SD rats. The cells were cultured in growth media containing Dulbecco’s modified Eagle’s medium/Nutrient Mixture F-12(Ham) (DMEM/F-12(1:1) basic(1X); Sigma) supplemented with 10% fetal bovine serum (FBS; Sigma). The cultures were maintained at 37 °C in a humidified 5% CO_2_ incubator. When the cells reached 80–90% confluency, about 5–8 days, cultures were harvested with Trypsin–EDTA solution (0.25% trypsin, 1 mM EDTA; Sigma). The media was changed every 2–3 days.

### Preparation of TAA

TAA (CAS No. 62-55-5, > 98.0% purity; Sang on Biotech, Shanghai, China) was dissolved in culture media (10 mg/mL), filter sterilized (0.22 um) then diluted to desired concentrations for use in specific assays as described below.

### Cell cytotoxicity assay (CCK-8)

CCK-8 assay was performed to assess the cytotoxic effect of TAA on BMMs. BMMs (2 × 10^3^ cells/well) were seeded into 96-well plates (Corning, USA) in a volume of 100 µL and cultured overnight. Then, the culture media was replaced by media containing different concentrations of TAA (0, 0.5, 1, 1.5, and 2 mg/mL), and each concentration was used in 5 parallel wells after adherence. The media was replaced with 100 μL of fresh media and 10 μL of CCK-8 solution (CCK-8; Beyotime Institute of Biotechnology, Shanghai, China) after culture for 12, 24, 36, 48, 60, 72, 80 and 96 h, the cells were then incubated for an additional 2 h, and the absorbance was measured at 450 nm with a microplate reader (Model 680; Bio-Rad Laboratories, Hercules, CA, USA). The cell viability and IC50 values were then calculated. The survival rate of BMMs (%) = experimental group A value/control group A value** × **100%. At least 3 independent experiments were performed.

### CFSE (5, 6-carboxyfluorescein diacetate succinimidyl oxy ester) assay

BMMs were cultured in different concentrations of TAA solutions (0, 0.5 and 1 mg/mL) for 24 h. Then, BMMs (1** × **10^6^ cells/mL) were labeled for 15–20 min at 37 °C with 3 μM carboxyfluorescein diacetate succinimidyl ester (CFSE; Cell Trace CFSE Cell Proliferation Kit, Invitrogen) in PBS supplemented with 0.1% BSA. CFSE-labeled cells were washed three times with PBS for 5 min at 400 g at room temperature and then cultured overnight. Then, the culture media was replaced by media containing different concentrations of TAA (0, 0.5 and 1 mg/mL). The cells were then incubated for 24 h. Cell fluorescence was evaluated by flow cytometry (Novo Cytec, ACEA, California, USA; 10,000 cells were analyzed in each sample), and the data were analyzed using Flow Jo 7.6 software. The division index (%) = the percentage of cells with reduced CFSE fluorescence intensity/the percentage of cells with constant CFSE fluorescence intensity. At least 3 independent experiments were performed.

### Cell cycle analysis

BMMs were exposed to TAA (0, 0.5 and 1 mg/mL) for 24 h. The cells were subsequently collected, washed with PBS and fixed with 75% ethanol overnight. The cells (1** × **10^6^ cells/mL) were then centrifuged for 5 min at 400 g, incubated with 10 mg/mL RNase and 1 mg/mL PI (Multi Sciences, CCS012) at 37 °C for 30 min away from light. Finally, the cell cycle distribution was analyzed by flow cytometry (Novo Cytec, ACEA, California, USA; 10,000 cells were analyzed in each sample). The percentage of cells in the G0-phase, the G1-phase, the S-phase, the G2-phase and the M-phase was analyzed by Cell Quest software (Becton Dickinson, Franklin Lakes, NJ). At least 3 independent experiments were performed.

### Wright–Giemsa stain and tartrate-resistant acid phosphatase (TRAP) stain

BMMs (1** × **10^6^ cells/well) were cultured with RANKL (50 ng/mL) and M-CSF (30 ng/mL) or varying concentrations of TAA (0, 0.5, and 1 mg/mL) in a 6-well plate for 7 days. Cells were fixed and stained with the Wright–Giemsa stain (C190805) and TRAP/ALP stain kit (code No. 294-67001), according to the instructions of the manufacturer. Osteoclasts were defined as TRAP-positive cells being stained purple under a light microscope. The number of TRAP-positive cells was analyzed by ImageJ software. At least 3 independent experiments were performed.

### Immunofluorescence

BMMs (1** × **10^6^ cells/well) were plated onto 35 mm glass-based dishes (801002; NEST Biotechnology, New Orleans, LA, USA) one day before stimulation with RANKL and M-CSF or TAA. Cells were then fixed in 4% polyformaldehyde for 30 min and permeabilized with 0.2% Triton X-100 for 15 min. They were then blocked with 5% BSA for 1 h. Primary antibody incubations were with the following antibodies: anti-tartrate resistant acid phosphatase (TRAP) (Abcam, ab191406, 1:1,000) in PBS containing 2% FBS overnight at 4 °C, followed by incubation with goat anti-mouse IgG H&L (DyLight488) (Abcam, ab96871, 1:400) in PBS containing 2% FBS as a secondary antibody for 2 h at room temperature. Finally, the cell nuclei were stained with DAPI (0.5 µg/mL) for 15 min, and images were obtained using a confocal fluorescence microscope. PBS was used for all washing steps. The number of TRAP-positive cells was analyzed by ImageJ software. At least 3 independent experiments were performed.

### Western blot

Cells were rinsed twice with PBS and lysed in RIPA lysis buffer (Beyotime Institute of Biotechnology), along with PMSF and Phosphatase inhibitor. The lysates were centrifuged, and the supernatants were collected. The protein was separated by 10% SDS PAGE and transferred to a polyvinylidene difluoride membrane. After blocking with skim milk (5%) in Tris-buffered saline and Tween-20 for 2 h at room temperature, the membrane was incubated overnight at 4 °C with the following primary antibodies: anti-PI3K (Abcam, ab151549, 1:1,000), anti-P-PI3K (Abcam, ab182651, 1:1,000), Anti-AKT1 (Abcam, ab81283, 1:10,000), Anti-P-AKT1 (Abcam, ab179463, 1:10,000), Anti-JNK1** + **JNK2** + **JNK3 (Abcam, ab18841-95, 1:1,000), Anti-P-JNK1** + **JNK2** + **JNK3 (Abcam, ab124956, 1:10,000), anti-P38 (Abcam, ab170099, 1:5,000), anti-P-P38 (Abcam, ab4822, 1:1,000), anti-Tartrate Resistant Acid Phosphatase (Abcam, Ab191406, 1:1,000), anti-cathepsin K (Abcam, ab19027, 1:1000), or anti-β-actin monoclonal antibody (Multi Sciences Biotech, Mab1445, 1:1,000). The membranes were then incubated with secondary antibodies conjugated to horseradish peroxidase (HRP), goat anti-mouse IgG, (GAM0072, Multi Sciences Biotech) or goat anti-rabbit IgG (GAR0072, Multi Sciences Biotech) for 2 h at room temperature. The membranes were then visualized using an ECL substrate kit (P1425; Multi Sciences Biotech) on the Omega Lum G Imaging System. Β-actin levels were used to standardize protein loading. ImageJ software was used to quantify band intensities. At least 3 independent experiments were performed.

### Flow cytometric analysis of apoptosis using Annexin V

The apoptosis of BMMs was analyzed by flow cytometry (Novo Cytec, ACEA, California, USA) using an Annexin V-FITC/PI apoptosis kit (Multi Sciences Biotech, AP101-100), according to the manufacturer’s protocol. Briefly, cells were washed twice with cold PBS and resuspended in 1**× **binding buffer at a density of 1** × **10^6^ cells/mL; 100 µL of the cell suspension was mixed with 5 µL FITC Annexin V and 10 µL propidium iodide (PI) and, then gently vortexed and incubated for 15 min at room temperature (25 °C) in the dark. Then, 400 µL of 1**× **binding buffer was added to each tube, and apoptosis was analyzed within 1 h. Cell fluorescence was evaluated by flow cytometry (Novo Cytec, ACEA, California, USA; 10,000 cells were analyzed in each sample), and the data were analyzed using Flow Jo 7.6 software. The data showed the percentage of apoptotic cells (Annexin V+ and PI+). At least 3 independent experiments were performed.

### Detection of intracellular Ca^2+^

The cells were exposed to different concentrations of TAA for 24 h. Then, PBS resuspended cells (1** × **10^6^ cells/mL) were incubated with 400 µL 3 µM Fluo-3 AM at 37 °C for 30 min, with vertical shaking every 5 min. The fluorescent intensity of Fluo-3 AM was measured using flow cytometry (Novo Cytec, ACEA, California, USA; 10,000 cells were analyzed in each sample) with excitation and emission wavelengths at 488 and 540–570 nm, respectively, and the data were analyzed using Flow Jo 7.6 software. The fluorescence intensity was proportional to the concentration of Ca^2+^. At least 3 independent experiments were performed.

### Statistical analysis

Statistical analysis was performed using Statistical Program for Social Sciences (SPSS) software 19.0. *T* test was used for statistical analysis among groups. Statistical analysis of all data was performed using GraphPad Prism 7 (GraphPad Software). *P < 0.05, **P < 0.01 and ***P < 0.001 were considered statistically significant. The results from at least three independent experiments were presented as the mean ± SD.

## Results

### Morphology and characterization of BMMs culture in vitro

**Fig. 1:**
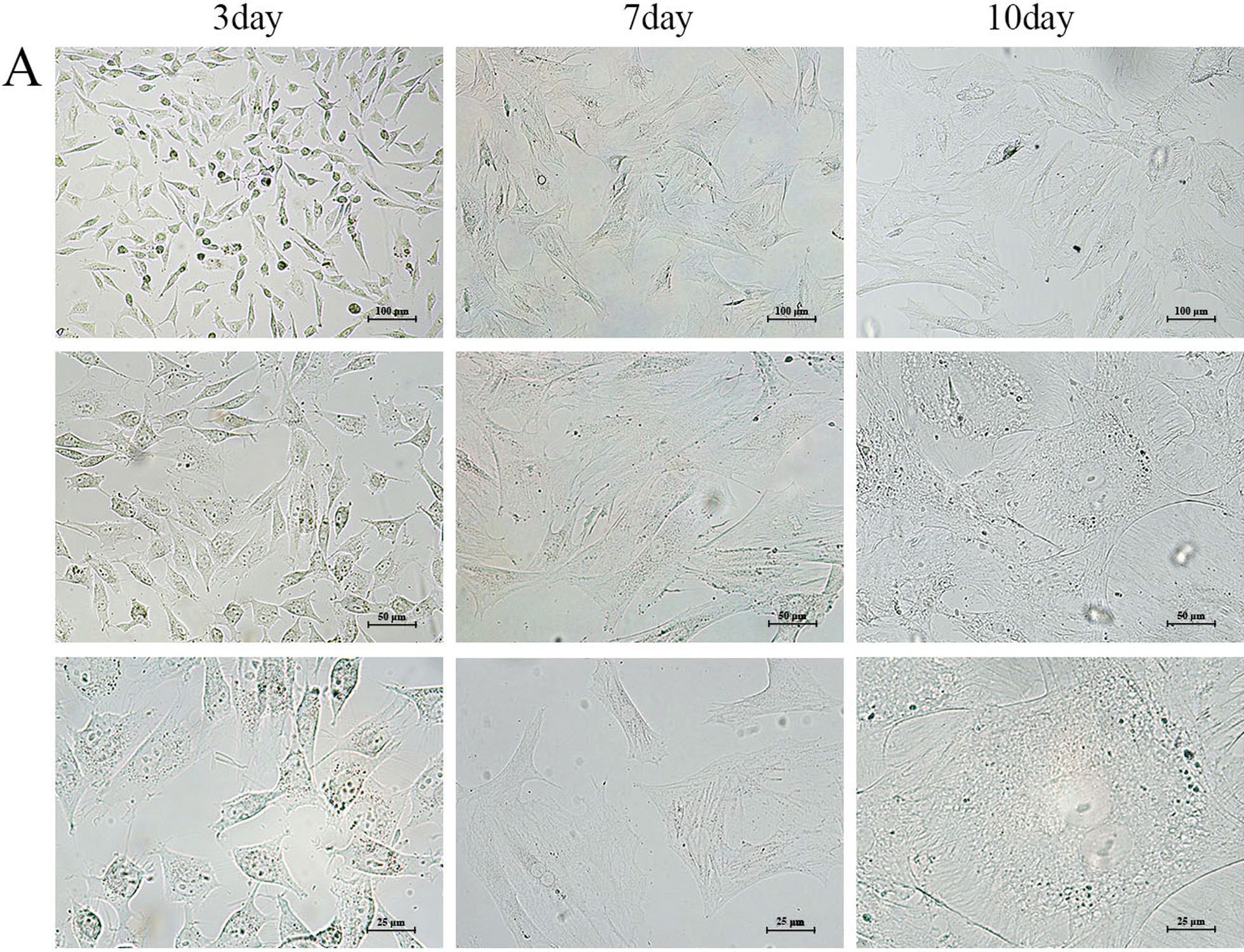
Characterization of Sprague–Dawley BMMs. **A** The cells were observed in the bright field, which grew into confluence after 3 day, 7 day and 10 day

We observed that freshly harvested BMMs were a uniform population; as cells continued to proliferate, the majority of cells became larger, and binuclear or even trinuclear can be seen. After culture for 7 days, the cell morphology changed gradually, the cell volume increased, gradually became irregular, radially adherent disk growth (Fig. 1A).

### BMMs proliferation and viability were inhibited by TAA

**Fig. 2:**
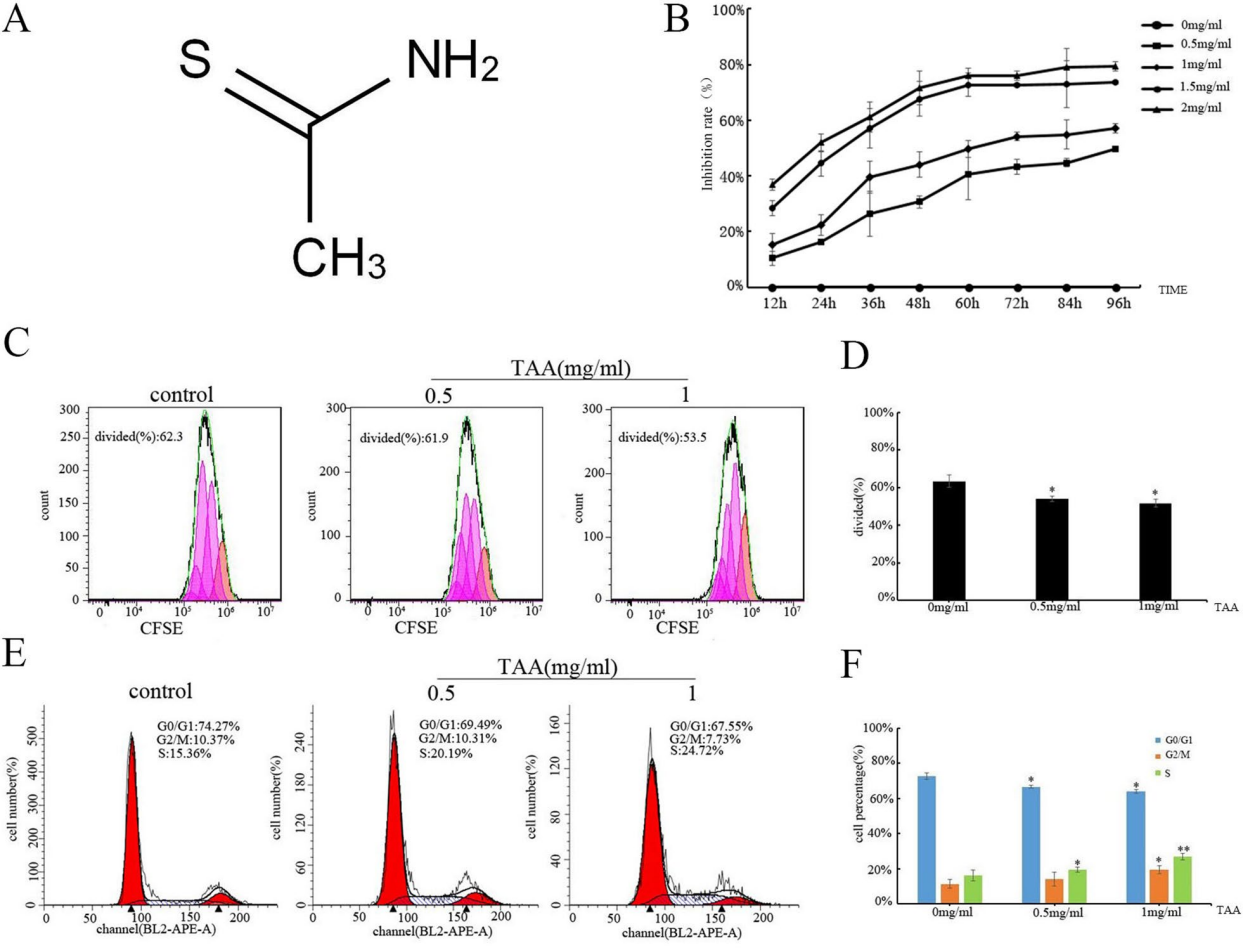
Effects of TAA on BMMs proliferation and viability. **A** Chemical structure of TAA. **B** Effects of TAA on the cell viability of BMMs. Cells were treated with the indicated concentrations of TAA for the indicated times. Cell viability was determined by the CCK-8 assay. Dates are expressed as mean ± SD. **C** BMMs were treated with the indicated concentrations of TAA for 24 h, then, cells were labeled for 15–20 min at 37 °C with 3 μM carboxyfluorescein diacetate succinimidyl ester (CFSE). Cell fluorescence was evaluated by flow cytometry. **D** The bar chart represented cells divided index. **E** Cell cycle distribution was measured in BMMs by flow cytometry analysis after TAA was treated with indicated concentrations for 24 h. **F** The bar chart represented the percentage of cells in G0/G1, S or G2/M phase, as indicated. Values are expressed as mean ± SD of three individual experiments; *P < 0.05 and ** *P* < 0.01 versus control

We then determined the inhibitory effect of TAA on BMMs using a CCK-8 assay upon exposure of BMMs with TAA at concentrations of 0, 0.5, 1, 1.5 and 2 mg/mL. Cell survival was found to be decreased in a dose and time-dependent manner, and cell viability reached a stable period after 72 h (Fig. 2B). The results showed that TAA reduced cell viability. Exposed to TAA at a dose of 1.5 and 2 mg/mL in 24 h, the cell viability was significantly decreased (to approximately 50%). In all the following experiments, 0.5 mg/mL and 1 mg/mL of TAA were selected for further investigation of the effects of TAA on BMMs and exploration of the related mechanisms.

We observed that the division index of 0.5 mg/mL and 1 mg/mL TAA-exposed BMMs decreased to 54.1% ± 1.5% and 51.8% ± 1.9%, respectively, compared to the control (63.2% ± 3.3%) after 24 h in TAA conditions (Fig. 2C, D, *P < 0.05). The results indicated that TAA could inhibit cell division.

To gain insight into the mechanism of TAA-induced growth inhibition in cells, we analyzed the TAA-dependent changes in the cell cycle distribution (Fig. 2E). Flow cytometric analysis showed that stimulation with TAA for 24 h caused a substantial increase in the ratio of S-phase cells relative to the whole cell population. The percentage of cells in S-phase was significantly increased to 19.4% ± 1.04% and 25.74% ± 1.77% in cells exposed to TAA at 0.5 mg/mL and 1 mg/mL, respectively, compared to 15.98% ± 1.16% in control cells (*P < 0.05). The percentage of cells in G0/G1-phase decreased compared to the control (67.6% ± 2.54%, 64.04% ± 3.05% vs. 72.78% ± 1.88%, *P < 0.05, **P < 0.01, shown in Fig. 2F).

### Osteoclastic differentiation of BMMs induced by TAA

**Fig. 3:**
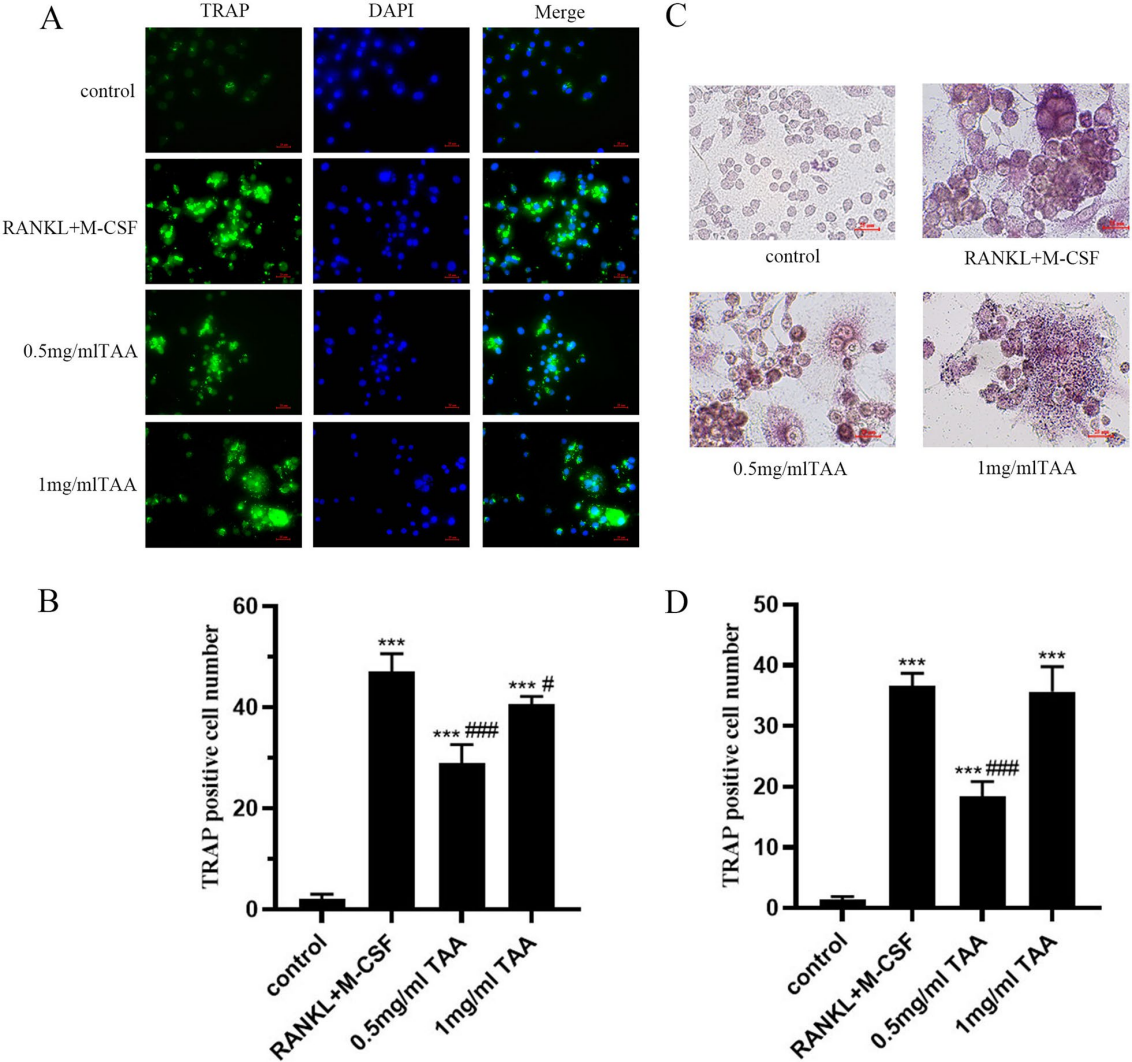
TAA promoted osteoclastogenesis in BMMs. **A** Representative images of immunofluorescent microscopy showing the TARP (green) and nuclear (blue) in RANKL and M-CSF-treated or TAA-treated osteoclasts. **C** Effects of TAA at different concentrations (0, 0.5, 1 mg/mL) on the formation of osteoclast-like cells derived from BMMs. **B**,** D** The number of osteoclasts showed TRAP-positive multinucleated cells (**B** for Immunofluorescence, **D** for TRAP staining). Values are expressed as mean ± SD of three individual experiments; *P < 0.05, **P < 0.01 and ***P < 0.001 versus control; ^#^*P* < 0.05, ^##^*P* < 0.01 and ^###^*P* < 0.001 versus RANKL and M-CSF

To investigate the effects of TAA on the osteoclastogenesis differentiation process of BMMs, immunofluorescence and TRAP staining were used to examine the osteoclast differentiation status of BMMs. We observed cell fusion leading to larger cells, the TRAP staining results of cells exposed to TAA for 7 days showed that the cells were irregular, with three or more nuclei (Fig. 3C). Also, BMMs induced with TAA began to show osteoclast-like morphology. After induced differentiation, TRAP staining results showed that there were few positive purple cells in the control group, while there were a large number of positive purple particles in the cytoplasm of the experimental group. The immunofluorescence results also confirmed this observation (Fig. 3A). There was almost no TRAP green fluorescence in the control group, while the TRAP fluorescence intensity in RANKL and M-CSF and TAA groups was significantly higher than that in the control group, and the higher the TAA concentration, the stronger the fluorescence intensity. Quantitative analysis showed that the numbers of TRAP-positive cells in the 0.5 mg/mL, and 1 mg/mL TAA groups were significantly higher than that in the control group (***P < 0.001 vs. control Fig. 3).

### Effects of TAA on PI3K/AKT pathway and osteoclast-specific protein expression

**Fig. 4:**
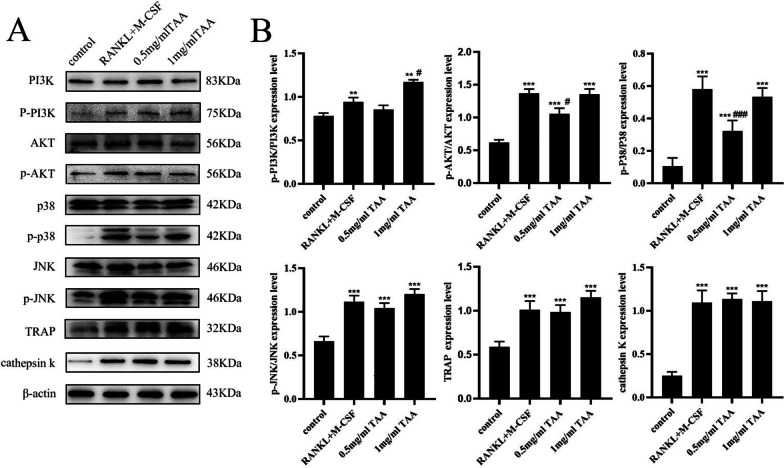
TAA promoted the expression of osteoclast-specific protein and PI3K/AKT pathway-related proteins. **A**, **B** Cells were cultured with TAA at the indicated concentrations for 7 days and lysed for western blot analysis with antibodies against PI3K, P-PI3K, AKT, P-AKT, P38, P-P38, JNK, P-JNK, TRAP, cathepsin k and actin. Values are expressed as mean ± SD of three individual experiments; *P < 0.05, **P < 0.01 and ***P < 0.001 versus control; ^#^*P* < 0.05, ^##^*P* < 0.01 and ^###^*P* < 0.001 versus RANKL and M-CSF

To investigate the effect of TAA on PI3K/AKT pathway and osteoclast-specific protein expression in BMMs, we used western blot analysis (Fig. 4). Western blotting showed that TAA exposure activated the protein expression of P-PI3K, P-AKT, P-P38, P-JNK, and promoted the expression of osteoclast-specific protein TRAP and cathepsin K. These results demonstrate that TAA exposure may promote osteoclast transformation of bone marrow cells by activating PI3K/AKT pathway-related proteins.

### TAA exposure increases intracellular Ca^2+^ and promotes apoptosis

**Fig. 5:**
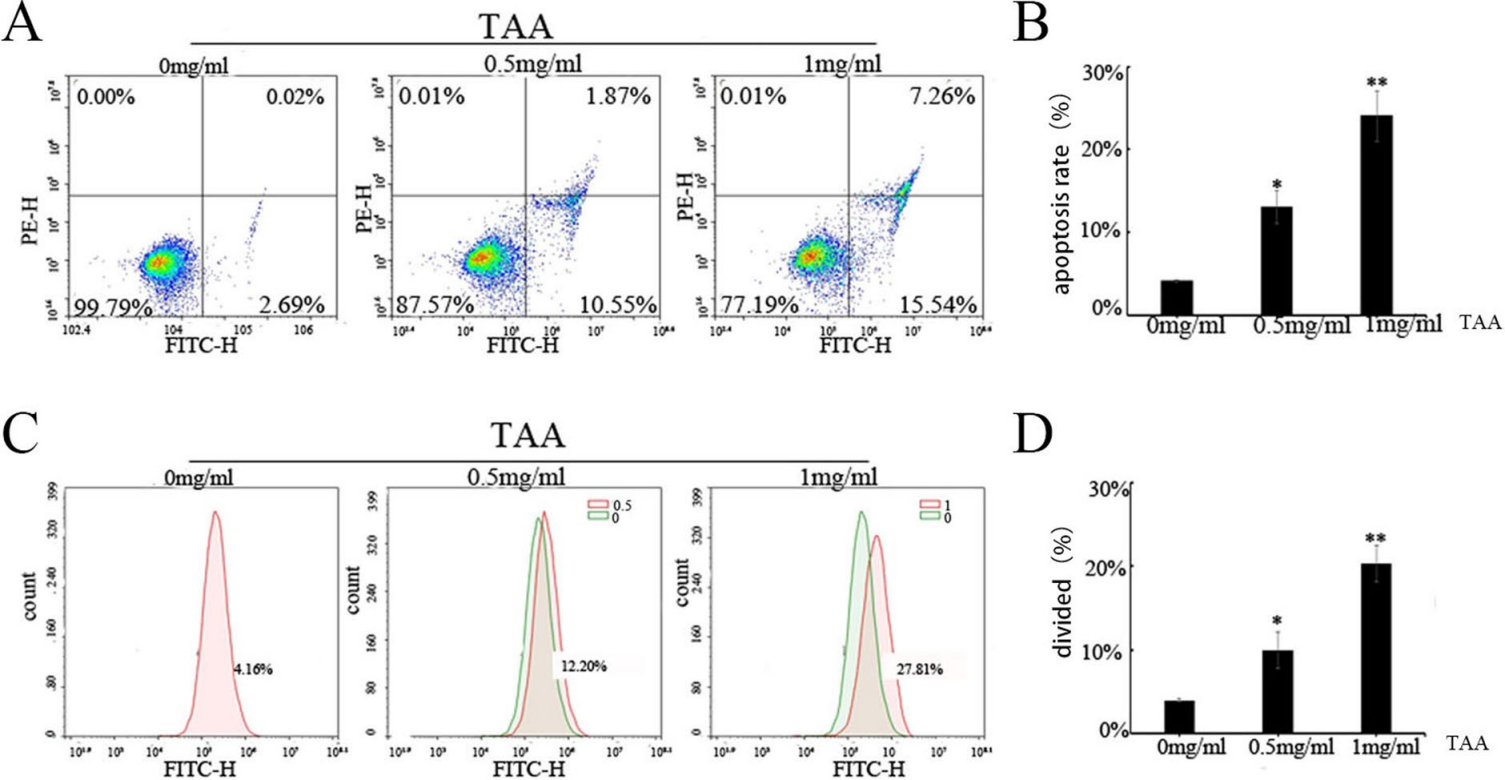
TAA exposure increases intracellular Ca^2+^ and promotes apoptosis. **A** Quantification of cell apoptosis by flow cytometry. BMMs were treated with TAA at the indicated concentrations for 24 h, then, cells were incubated with FITC Annexin V in a buffer containing propidium iodide (PI) and analyzed by FCM. **B** Statistical bar graph showing the apoptosis ratio. **C** Analysis of intracellular Ca^2+^ in BMMs. Cells were treated with TAA at the indicated concentrations, then the cells were incubated with 400 μL 5 μM Fluo-3 AM at 37 °C for 30 min. The fluorescent intensity of Fluo-3 AM was measured by the flow cytometry analysis at 488 nm (excitation) and 540–570 nm (emission). **D** The bar chart represented the intensity of fluo-3 AM in cells. Values are expressed as mean ± SD of three individual experiments; *P < 0.05, **P < 0.01 and ***P < 0.001 versus control

Next, we explored whether TAA induced BMMs apoptosis. Figure 5A, B shows that, as the concentration of TAA increased, the percentage of apoptotic BMMs increased. To further explore whether TAA was a calcium channel blocker, intracellular Ca^2+^ was detected by evaluating the fluorescent intensity of Fluo-3 AM. As shown in Fig. 5C, D, as the TAA concentration increased from 0 to 0.5 mg/mL and 1 mg/mL, the fluorescent intensity of Fluo-3 AM increased from 4.16 to 12.2% and 27.81%, respectively. The results indicated that BMMs intracellular Ca^2+^ were increased upon exposed to TAA.

## Discussion

Overactivation of bone resorption plays a critical role in the pathological mechanisms of osteoporosis [34]. Osteoclasts, as the unique multinucleated cells that can dissolve bone matrix, are derived from the monocyte/macrophage lineage of hematopoietic precursors [35]. It has been confirmed that these progenitor cells fuse to form osteoclasts under the effect of RANKL and M-CSF.

Previous studies have found that propranolol can promote osteoclastogenesis of mesenchymal stem cells (MScs) while inhibiting their proliferation, explaining that cell proliferation and differentiation can influence each other [36]. TAA is a potent experimental hepatotoxin and hepato-carcinogenic compound that is often used to induce fulminant hepatic failure in experimental animal models [37]. Our research shows that TAA has a toxic effect on BMMs, and can inhibit the proliferation of BMMs in a dose- and time-dependent manner in vitro. TAA can even promote the apoptosis of BMMs, which may be caused by the increase in intracellular calcium. More importantly, we show for the first time that TAA may be directly related to osteoclastogenesis and can induce BMMs to differentiate into osteoclast-like cells. 0.5 or 1 mg/mL TAA has less toxic effects on BMMs, because its main effect on BMMs may be to induce osteoclast differentiation, inhibit cell proliferation during the differentiation process, and promote cell apoptosis.

P2X7/PI3K/AKT pathway has been shown to regulate osteoclast survival and differentiation. Ma [38] found that the suppression of PI3K/AKT signaling was further inhibited osteoclast and osteoblast differentiation after long-term Cd exposure. Under the stimulation of RANKL and M-CSF, PI3K/AKT pathway proteins are activated in osteoclast precursor cells and modulate osteoclastogenesis and osteoclast activity [39–42]. 0.5 or 1 mg/mL TAA can promote osteoclast differentiation, and the effect is similar to RANKL and M-CSF. Same as the previous reports, our results showed that TAA promoted the expression of p-PI3K, p-AKT, p-P38 and p-JNK, indicating that it may promote osteoclast differentiation by activating the PI3K/AKT pathway (Fig. 4).

In conclusion, our data show that TAA affects the proliferation of BMMs, arrests them in S-phase and promotes their apoptosis. But the most important finding is that 0.5 or 1 mg/mL TAA may promote the osteoclast transformation of BMMs by activating the PI3K/AKT pathway, and promoting the expression of osteoclast-specific proteins TRAP and Cathepsin K, which is equivalent to RANKL and M-CSF. So, we hypothesize that TAA may have a similar effect to RANKL and M-CSF, which can induce the formation of osteoclasts. RANKL and M-CSF are expensive and not readily available, while TAA may be an inexpensive alternative. The potential of TAA to induce osteoclasts suggests that we may have found a cheap osteoclast inducer. In order to further prove the effect of TAA on osteoclast differentiation, current efforts are directed at evaluating the effects of TAA on osteoclast differentiation in vivo.

## Data Availability

The data that support the findings of this study are available from the corresponding author upon reasonable request.

## Abbreviations

TAA: Thioacetamide
BMMs: Bone marrow macrophages
M-CSF: Macrophage colony-stimulating factor
RANKL: Receptor activator of nuclear factor kappa B ligand
